# Tracking Infant Development With a Smartphone: A Practical Guide to the Experience Sampling Method

**DOI:** 10.3389/fpsyg.2021.703743

**Published:** 2021-12-06

**Authors:** Marion I. van den Heuvel, Anne Bülow, Vera E. Heininga, Elisabeth L. de Moor, Loes H. C. Janssen, Mariek Vanden Abeele, Myrthe G. B. M. Boekhorst

**Affiliations:** ^1^Department of Cognitive Neuropsychology, Tilburg University, Tilburg, Netherlands; ^2^Department of Psychology Education & Child Studies, Erasmus University Rotterdam, Rotterdam, Netherlands; ^3^Department of Developmental Psychology, Tilburg University, Tilburg, Netherlands; ^4^Department of Developmental Psychology, Groningen University, Groningen, Netherlands; ^5^Department of Quantitative Psychology and Individual Differences, KU Leuven, Leuven, Belgium; ^6^Department of Youth and Family, Utrecht University, Utrecht, Netherlands; ^7^Department of Clinical Psychology, Leiden University, Leiden, Netherlands; ^8^imec-mict-UGent, Department of Communication Sciences, Ghent University, Ghent, Belgium; ^9^Department of Medical and Clinical Psychology, Tilburg University, Tilburg, Netherlands

**Keywords:** experience sampling method/ecological momentary assessment, infant development, longitudinal data, ambulatory monitor, infancy

## Abstract

The COVID-19 pandemic has forced developmental researchers to rethink their traditional research practices. The growing need to study infant development at a distance has shifted our research paradigm to online and digital monitoring of infants and families, using electronic devices, such as smartphones. In this practical guide, we introduce the Experience Sampling Method (ESM) – a research method to collect data, in the moment, on multiple occasions over time – for examining infant development at a distance. ESM is highly suited for assessing dynamic processes of infant development and family dynamics, such as parent-infant interactions and parenting practices. It can also be used to track highly fluctuating family dynamics (e.g., infant and parental mood or behavior) and routines (e.g., activity levels and feeding practices). The aim of the current paper was to provide an overview by explaining what ESM is and for what types of research ESM is best suited. Next, we provide a brief step-by-step guide on how to start and run an ESM study, including preregistration, development of a questionnaire, using wearables and other hardware, planning and design considerations, and examples of possible analysis techniques. Finally, we discuss common pitfalls of ESM research and how to avoid them.

## Introduction

The COVID-19 pandemic made traditional observational and experimental methods unavailable due to closing labs and the introduction of strict rules regarding physical contact. This forced infant developmental researchers to rethink their traditional research practices by using novel paradigms and electronic devices. One way of studying infant development without physical contact is to use the Experience Sampling Method (ESM) – also called Ecological Momentary Assessment (EMA), Ambulatory Assessment (AA), or Mobile Experience Sampling Method (MESM, m-ESM) – a research method to collect data, in the moment, on multiple occasions over time (Csikszentmihalyi and Larson, 1987; Stone and Shiffman, 1994). In infant research, this often takes the form of the parent filling out questionnaires about their infant’s behavior and mood, tracking their infant’s sleep or activity patterns, and/or reporting on their own mood, thoughts, practices, and behaviors.

The first ESM-type studies, from the 1980s onwards, have provided unique insights into daily life processes by using paper-and-pencil questionnaires or “diaries.” Infant researchers mainly used diary measures in which mothers and fathers kept track of their infant’s activities, such as sleep (St James-Roberts and Plewis, 1996) or cry and fuss behavior (Barr et al., 1988; Fujiwara et al., 2011). To date, diary work is still used to measures infant sleep and crying behavior and is often even considered more valid than using one-time questionnaires (Teti et al., 2010; Fujiwara et al., 2011; Hechler et al., 2018; Bacaro et al., 2020). Despite the strong tradition in diary work in infant research and the large overlap in methods between diary work and ESM research, the use of ESM remains sparse in infant research. With the COVID-19-infused shift in research practices, there may be momentum to welcome ESM to the skillset of infant researchers and use it innovate the field.

Over the years, researchers have provided systematic guides and overviews of ESM methodology to discuss the various steps that are important to consider when setting up an ESM study (e.g., sample size calculation, software, implementation, and data analysis) (Christensen et al., 2003; Uy et al., 2009). Even though these studies have provided an important outline of ESM research, they were conducted prior to the regular use of the smartphone (e.g., using computerized methods, Personal Data Assistants (PDAs), and paper-and-pencil methods). Over the recent years, the daily use of smartphones has become more common. As a result, many ESM applications and software for smartphones have been developed. Smartphones are therefore a major step forward in ESM research, allowing for more accurate “in-the-moment” collection of data. Thus, we build on this previous literature by specifically catering to developmental researchers and by providing a more grounded overview and step-by-step guide of ESM research for smartphone monitoring. The aim of this practical guide was to provide infant researchers with an introduction to ESM research for studying infant development and to lower the potential barrier for including this technique into their skillset. We start this practical guide by explaining what ESM entails and by discussing advantages and disadvantages for using ESM in infant research. Next, we provide a brief step-by-step guide on how to design and run an ESM study, including preregistration, selection/development of questionnaires, design planning, running the study, and analysis techniques. Finally, we discuss common pitfalls of ESM research and how to avoid them.

## Esm in Infant Research

### What Is ESM Research?

With ESM, participants fill out micro-surveys several times a day for several consecutive days. ESM captures “Life as it is lived,” by assessing participants’ cognitions, emotions, and experiences several times a day in the context of daily life (Bolger et al., 2003; Scollon et al., 2003). Note that a diary study could also be considered as ESM, when it asks parents to make diary entries multiple times a day, such as every half hour or after each nap of their infant (e.g., Barr et al., 1988). However, many diary studies require participants to recall events, feelings, and/or thoughts only once a day (i.e., daily diary), for example, before going to bed. Additionally, ESM research nowadays is often conducted with the use of a smartphone or other electronic device and can be accompanied by other technologies (e.g., activity watch, heart rate sensor, and/or beacon).

### Infant Research Using ESM

Experience sampling method studies that investigate infant development (up to the age of 24months) and family dynamics can include parental-related measures (e.g., maternal mood and feeding practices), infant-related measures (e.g., crying and sleep), or a combination of both. Only very few studies so far have incorporated ESM to study these measures. One example is a study by Sawada et al. (2015), that included ESM to assess daily maternal reports of infant fussing and crying at 12months postpartum. They studied associations between maternal stress (conceptualized as “felt security”) at 6months postpartum and infant fussing and crying at 12months postpartum in mother-infant dyads with a healthy infant (N=93) and dyads with infants who have a medical problem (N=42). Infant fussing and crying was measured with ESM by paging the mothers with a personal digital assistant (PDA) three times a day for 7 days. Among dyads with an infant born with medical problems, higher felt security of the mother predicted decreased fussing and crying of the infant, but not among dyads with a healthy infant. Another study used ESM to track soothing behaviors of 157 mothers after an intervention that focused on reducing the use of feeding to sooth an infant (Adams et al., 2019). The mothers reported on infant fussing and crying with 4-h intervals, between 10AM and 10PM, and filled out a morning and evening questionnaire. They found that the parenting intervention was successful in reducing the use of food to soothe and increased the use of alternative soothing strategies in response to infant fussiness. With this ESM technique, the authors were able to gather detailed, ecologically valid data on mothers’ soothing techniques, right after soothing took place.

Another research group recently used ESM to follow pregnant mothers with a substance use disorder to examine momentary fluctuations in posttraumatic stress disorder (PTSD) symptoms, prenatal bonding, and substance craving (Sanjuan et al., 2020). The pregnant women filled out three 5- to 10-min questionnaires for 28days asking about PTSD symptoms, prenatal bonding (i.e., the quality of maternal affective experience and intensity of preoccupation with fetus), and substance cravings. Results showed that higher momentary ratings of PTSD symptoms in these mothers were associated with lower quality (but not intensity of preoccupation) of prenatal bonding, which in turn was associated with greater craving for substances. Next, another study used ESM to capture maternal experiences and emotions in the context of real-world, day-to-day parenting challenges (Hajal et al., 2019). For this study, mothers (N=55) were interviewed over the phone four times a day for 6days about their momentary emotions, motivational states, and parenting behaviors. They found that maternal reported momentary emotions were more consistently associated with momentary motivational states (i.e., desire to approach/engage and avoid/disengage with their infant) than reported behaviors and underscore the importance of momentary emotions in studying family dynamics.

Finally, a recent study used ESM to assess the relationship between testosterone and time fathers invested in their infants (Corpuz et al., 2021) In this study, testosterone was measured in first-time fathers (N=225) during their transition into parenthood (pregnancy, 3months postpartum, and 9–10months postpartum). For the ESM part of the study, the authors assessed the time invested in direct infant care. To measure this, participating fathers received eight text messages for 6days that they were not working. The study found a relationship between accelerated testosterone rebound (increase) and less time spent with their infant. There was also a positive association between testosterone rebound and the quality of care fathers showed (not measured using ESM).

In sum, while ESM research in infancy is sparse, the summarized studies show the feasibility of using ESM in pregnant women and early postpartum mothers (even when suffering from PTSD and drug abuse) to assess dynamic and momentary processes regarding infant and maternal mood, as well as infant-parent interactions and father involvement in infant care. However, many research questions in infant development on family dynamics remain unexplored. ESM could add potential new insights given its advantages over experimental and questionnaire research, allowing for momentary assessment of the infant-parent dynamics and frequent fluctuations of mood and behavior.

### Advantages of ESM for Infant Research

The most notable advantage of ESM is that it enables researchers to capture data “*in situ”* (Naab et al., 2019). With ESM, researchers can gain information not only about *content*, but also its *context* (Hektner and Csikszentmihalyi, 2002). Because ESM sheds light on the “situatedness” of human experience, it is a highly suitable method for studying context-dependent processes that occur during infant development (Bamberger, 2016). For instance, researchers can assess whether an infant is happy or sad and link that mood to contextual information, such as their sleep quality or maternal mental health. To date, such an analyses have not yet been conducted in infancy research. For an example in adolescent research, see Kim et al. (2018).

A second advantage of ESM is that it offers a possibility to assess complex phenomena, such as infant development and family functioning in an *ecologically valid way* (Trull and Ebner-Priemer, 2009) and with reduced recall bias (Schwarz, 2007). Recall bias refers to the bias that arises when people retrospectively report on behavior, emotions, or cognitions, as is typical in cross-sectional or retrospective longitudinal survey research. ESM reduces recall bias because it requires no or a very limited retrospective recall (Scollon et al., 2009). An alternative to reduce recall bias in self-reports would be to directly observe parents and children in lab settings. Such observations are costly, however, limited in time, do not give access to mental states or cognitions, and lack ecological validity. The latter may be solved by observing in the home or in public settings, but this requires extensive consideration of research ethics, and such observations may also influence the ecological validity as parents behave differently when being knowingly observed (Vanden Abeele et al., 2020).

Third, because ESM enables multiple assessments over a relatively short time span, researchers can collect data about phenomena that are potentially short-lived or transient in nature and, thus, allows researchers *to capture the dynamic nature of events* and shed light on how events unfold in everyday life (Hektner and Csikszentmihalyi, 2002). These events may concern one individual; for instance, researchers can explore whether an infant’s lack of sleep predicts subsequent crying behavior. Additionally, ESM is also suited to study family dynamics (Larson et al., 1996; Repetti et al., 2015; Bamberger, 2016). Especially relevant in this regard are studies that collect dyadic data (e.g., parent-infant data), for instance, to assess whether there are transmission effects in parents’ and infants’ emotional states.

A fourth advantage of ESM is that the examination of temporal patterns offers the possibility to investigate not only between-person, but also *within-person* (or *within-family*) *processes* (Scollon et al., 2003; Keijsers and van Roekel, 2018). This is of great importance in the study of infant development. It is thinkable that group-level associations between parenting behaviors and child development do not uphold when examining within-family associations. For instance, while there may be a positive within-person association between infant sleep duration and infant mood, the between-person association may be absent or even reversed. In other words, it is reasonable to expect that an infant that sleeps longer will be more rested and thus have a better mood (within-person effect). However, it may not be the case that infants that (on average) sleep longer are generally more happy (between-person effect).

A fifth advantage of ESM is that infant development can be studied solely at a distance. This research method utilizes data collection through smartphone, allowing for complete digital monitoring. This is especially important during the COVID-19 pandemic, when observational and experimental research methods were unable to be used due to lockdown regulations (e.g., closed labs and contact restrictions). Apart from during the COVID-19 pandemic, it is also important to mention that research from a distance can be of added value for infant research in general. ESM research would limit travel time, which would be advantageous for parents living further away and infants in general. It can be incorporated in the daily life of parents with young infants, taking parenting schedules into consideration.

### Disadvantages of ESM for Infant Research

Assessing parent or infant behavior several times a day also has several disadvantages. It can be *burdensome for parents* (Eisele et al., 2020). With ESM, parents usually receive multiple short questionnaires per day. It is not uncommon for ESM studies, for instance, to administer even up to 8–15 short questionnaires per day (e.g., van Roekel et al., 2019; Dietvorst et al., 2021). Although completing each questionnaire usually takes no more than a few minutes, participation in an ESM study can be demanding for young parents, both in terms of the total time investment and in terms of the (cognitive) resources that are needed to always be able to respond to the questionnaires in a timely and qualitative manner (Sonnenberg et al., 2012).

Because of the burden associated with ESM studies, another significant disadvantage of ESM is *self-selection bias:* Parents who can better cope with the burden of the ESM may be overrepresented in the sample, and this may lead to an overrepresentation of participants in the sample with relevant characteristics, such as greater motivation (Scollon et al., 2009). This disadvantage can be highly relevant for infant research. For parents of young children who already struggle to manage the day-to-day organization of their household or those parents who experience heightened levels of psychopathology, participation in an ESM study may be especially burdensome. This becomes specifically problematic when inclusion of specific subgroups is of interest to the researchers.

A third disadvantage of ESM is potential *bias in the responses*. Since ESM requires the parent to have access to a device (usually their smartphone) to answer questions, not all activities are easy to capture with this technique. For instance, when a parent goes swimming with their infant, they will probably not check their phone, and therefore, an ESM study that focuses on parent–child quality time may miss instances of such activities. Furthermore, in specific subgroups of individuals, such as parents with mental illness, this bias may also play a role. For example, in their guide, Palmier-Claus et al. (2011) mentioned that altered sleep patterns (e.g., unusual waking/sleeping times) are important to consider in individuals with mental illness, as they may therefore not always be able to respond to the ESM triggers. Additionally, by its very nature, ESM will interrupt parents in their daily activities. Especially in the context of studies focusing on infant development, inducing such interruptions for the sake of scientific research comes with ethical considerations. Recent studies showed that mobile device use makes parents up to five times less responsive to young children’s bids for attention (Vanden Abeele et al., 2020). Hence, researchers carry a responsibility when asking parents to participate in an ESM study.

## Practical Step-By-Step Guide

**Figure 1:**
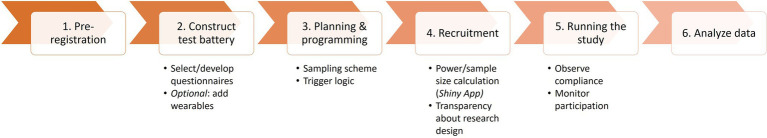
Overview of the steps for planning and running an Experience Sampling Method (ESM) study.

An overview of the different steps that are important to consider when planning and running an ESM study is provided in Figure 1.

### Preregistration of an ESM Study

Preregistration of ESM studies is highly recommended. Preregistration is a specification of the research plans in advance of the study and prevents the unintentional use of the same data to both generate and test a hypothesis. Separating exploratory (i.e., hypothesis generating) from confirmatory research (i.e., hypothesis testing) improves the quality and transparency of research. Preregistration of an ESM study has many of the same components as other (laboratory-based) studies of infant development, but also include additional elements, such as sampling scheme, trigger logic, and monitoring strategies (for explanation, see section *planning and programming an ESM study*). In a recent paper by Kirtley et al. (2021), the process of preregistering an ESM study is described and an open-access template is provided.

### Developing an ESM Questionnaire

The next step is to create a test battery for the ESM study by selecting instruments. It is important that the instruments used in an ESM study cater to the format of micro-surveys that are administered several times a day and that they target the momentary experiences of participants. In contrast to most single assessment questionnaires, ESM items should accommodate momentary states, making it clear that the question is regarding experiences “*right now”* or refers to the time *in between* assessments (Myin-Germeys et al., 2018). Some studies have addressed these aspects by adjusting existing questionnaires (e.g., shortening or rewording) that are commonly used for the retrospective design. Nonetheless, different types of questions can be applied to ESM research. In their paper, van Berkel et al. (2017) draw on common ESM question types, addressing the usage and challenges of different types (e.g., checkboxes, text field, sliders, Likert scale, and photos). ESM questions can also be organized in a branching structure to decrease the participants’ burden (e.g., using a checklist with multiple answer options and only show the follow-up Likert scale questions for the items selected by the participant). The ESM item repository^1^ can facilitate in construction of an ESM-friendly test battery.

To date, there are very few items/questionnaires available that are tailored to infant development and family dynamics and practices, such as feeding practices, sleeping, and mother-infant interaction. An example of an item suitable to assess infant mood within an ESM design could be: “how would you rate your child’s mood” (0=very fussy to 5=very happy) (Mindell and Lee, 2015). Furthermore, very recently, an ESM questionnaire was developed by the first and last authors (MvdH and MB) to measure maternal baby-related anxiety, the “Baby-related Anxiety and Behavior Inventory (BABI).” The questionnaire and development protocol can be downloaded at OSF (Boekhorst et al., 2021, February 18^2^).

When developing an ESM questionnaire battery, it is essential that the participants’ burden is also taken into consideration (Consolvo and Walker, 2003). As addressed by Myin-Germeys et al. (2018), an ESM questionnaire should only take 2min or less to complete and shorter questionnaires lead to higher compliance, especially considering the already busy schedule of young parents raising an infant (see section compliance). Furthermore, it is also important to make sure that the constructs measured are variable (enough) so participants’ responses to the recurring questionnaires are not always the same. For instance, ask about behaviors, thoughts, and mood states, instead of more static features, such as opinions, knowledge, and personality. Finally, it is also advised to pilot the questionnaire to estimate psychometric properties, the time it takes, and ask for feedback on feasibility, the likability of the questionnaire, and parents’ personal experience.

While this article mainly focuses on ESM as a quantitative research method, it is relevant to note that there are also alternative, more qualitative, approaches to ESM. Kaufmann and Peil (2019), for instance, developed the Mobile Instant Messaging Interview (MIMI) method, in which they use a mobile messenger, such as WhatsApp, to interact with participants at different time points during the day. With the MIMI technique, the researchers, for instance, start a conversation over WhatsApp asking whether the participant is using any social media at that moment by asking the participant to send a description, photo, and/or video. Another example is a recent study by Cho and Ilari (2021), that investigated the association between child mood, music, and parenting during the COVID-19 pandemic. The authors shared music videos with the mothers (to play for their child) and received video and photo materials back from the participants. Adding qualitative items can be valuable in the context of infant development research, as they enable researchers to capture data that is rich in nature. A major drawback of qualitative measures, however, is the time investment of the participant and, in some cases (e.g., sending photos), privacy concerns.

### Using Smartphones

The daily use of smartphones has become more common, also allowing researchers to use them to collect data for ESM data collection. As a result, applications have been specifically designed for ESM studies using smartphones. The use of smartphones for ESM research has several advantages. Using smartphones allows for a more efficient implementation of the questionnaires that are completely online (e.g., without needing to use any paper-pencil questionnaires), the administration is more sufficient for researchers, data collection, and completion is more time-efficient, and ESM applications send automated triggers (e.g., at random, scheduled) to the participants’ smartphone, which in turn can help with compliance (Ainsworth et al., 2013; Thomas and Azmitia, 2015; Stieger and Reips, 2019). Using smartphones for ESM data collection are also efficient for infant researchers as parents receive an automated reminder on their smartphone without needing to schedule questionnaire completion themselves, which may be more difficult during parenting practices of an infant (e.g., caretaking, play, feeding).

As the number of ESM studies grow steadily, new mobile applications and software packages are being developed, with a wide range in technical features and pricing. Researchers can consider several things when selecting the ESM software or applications. For example, is working with an application *via* smartphone necessary or would setting up a study in a web tool be sufficient? The latter would enable participants to use multiple devices to answer surveys or include participants without smartphones. However, participants would not be able to answer surveys when they are offline and the study cannot collect additional passive data. Systems also differ in customizability. In some Open Source cases, it is possible to customize the platform entirely, but this might take getting used to the system. When using a mobile application, it is important to consider the possible operating systems. Some only work on Android and others also partly on iOS, but there are also applications who work properly on both operating systems. Although most companies provide a limited free trial or plan, they often use different ways of payment (e.g., payments for a certain amount of time, or payment per participant). Various Open Source platforms are free for all users, while others provide additional licenses for extra features.

Nonetheless, it is important to note that ESM applications are developing rapidly, suggesting that personal research on the possibilities and costs of different applications is essential. From personal experience, companies are usually open to suggestions for adding features to expand their applications or packages. This does not always have to result in paying additional costs, depending on the amount of similar suggestions they receive.

### Using Wearables and Other Hardware

Traditionally, ESM studies rely on participants answering questionnaires, but the use of smartphones enables researchers to also passively collect data on, for instance, health and social activities (i.e., location, accelerometer activity, device usage data, and microphones). Moreover, some applications even allow a seamless integration with wearables for tracking activity, sleep, physiological arousal, or location (Trull and Ebner-Priemer, 2009). Therefore, the next step is to decide whether or not to include wearables or other hardware to the ESM study. Here, we discuss two types of hardware that can be especially useful for infancy research: activity trackers and beacons.

#### Activity Trackers

To get more insight into, for instance, infant sleeping behavior (i.e., amount of sleep, quality of sleep), it is possible to include an actigraphy monitor (i.e., ActiGraph and Fitbit) in an ESM study. This additional information can help to answer validation questions, such as whether the measured sleep and nap time by the ActiGraph corresponds to maternal self-report (Tikotzky and Sadeh, 2009). Infants typically wear the (mini) ActiGraph on their ankle while they sleep. Such devices can also be used to decrease the burden of asking parents to report on infant sleeping patterns by measuring it (with even more precision) directly.

#### Beacons

While ESM studies with self-report items of questionnaires can already contribute to a better understanding of the parent-infant relation, gaining even more insight by using an unobtrusive measure to assess actual parent–child proximity in daily life seems promising. Asking parents about the amount of time they spend with their infant can still be biased or influenced by nonresponse. Instead, fine-grained information on parent-infant proximity (i.e., distance in meters) can be obtained when using Bluetooth and Bluetooth beacon devices. When the beacon is placed in the crib or sewn into clothing, researchers can measure the exact times that a mother is close to her infant. Researchers can configure the beacon devices with specific settings (i.e., time interval of checking for other beacons, appropriate distance between beacons) and give a beacon device to each member of a family (i.e., mother, father, and infant) to study family dynamics. Another option is to trigger a questionnaire or another form of data collection, such as audio recording, with beacon information. A very recent example of this is the pilot study by Salo et al. (2021), in which the researchers developed clothing for infants with technology in it (i.e., a beacon and audio recorder) – called the “TotTag.” The audio recorder was triggered and started recording when the mother (who was also wearing a beacon) was close to the infant.

### Planning and Programming an ESM Study

Naturally, the design of the ESM study should align with the aims and research questions. When setting up an ESM study, several factors should be taken into consideration, such as the type of sampling and intervals between assessments. Sampling can be time-based, meaning that participants will receive a notification on fixed or random times as programmed by the researcher. Assessments can also be event-based. In this case, participants have to initiate the completion of an assessment after an event has occurred (such as after feeding or infant naps). Event-based assessment can also be triggered automatically, for instance, when a participant is at a certain location (GPS-based or beacon, see above at *beacons*; e.g., when parents are at home *with* the infant or when they are at work *without* the infant). Importantly, for measuring social situations, such as interactions, the time-based and event-based sampling designs lead to comparable data quality (Himmelstein et al., 2019). After deciding whether event-based and/or trigger-based works best for the purpose of a study, the next two important factors to consider are the sampling scheme and the trigger logic.

#### Sampling Scheme

When using time-based sampling, it is important to consider issues, such as the time window in which the sampling will occur. When the study aim is regarding parent-infant activities, it is important to take into account whether parents go to work or are otherwise not with their infant (e.g., daycare), but when examining mood over time, it is important to assess mood several times throughout the day since mood fluctuates constantly. When sampling during the night, participants will likely miss several assessments (and it can be very burdensome). When interested in nighttime activities and/or interactions, it is advised to very carefully inform participants and/or ask about nighttime in the morning (e.g., how well did you sleep? and How did the infant sleep?). In some studies, it might be convenient to personalize the sampling scheme to increase compliance, while in other studies, this could affect the outcomes (e.g., data on parental stress/mood during the day might remain unnoticed or inaccurate if questionnaires are only received when it is better suited for the parents, such as during infant naptime or during a day off). Important to note is that not all software applications provide the opportunity to personalize schemes. Additionally, for some analyses, the time interval between the measurements needs to be equal. Therefore, planning analyses before designing the study is advised.

#### Trigger Logic

Most software applications allow for decisions on a trigger logic, for example, when the questionnaires are prompted and for how long participants can respond (e.g., time-based: trigger questionnaire between 9 and 10:A.M, event-based: trigger when beacon signals contact with infant). Participants can also be reminded to fill out unanswered questionnaires by sending them notifications (most applications allow this). Notifications can be triggered with a logic, for instance after 30min of not completing the questionnaire. Usually, questionnaires will be inaccessible after a certain time (expired) or when the next questionnaire is prompted.

### Recruiting Parents for Your ESM Study

Before recruiting parents for the ESM study, it is advised to calculate an ideal sample size given the analysis plan and desired power. While power analyses are often used to inform sample size planning in general (Cohen, 1988), they are not yet well-established in ESM research. This is mainly because the multilevel structure of the data (i.e., many nested measurements per participant) makes power calculations challenging (de Jong et al., 2010; Bolger, 2011). Recently, however, Lafit et al. (2021) published a new “*Shiny App*” that helps to perform simulation-based power analyses. Still, pilot data are necessary to be able to perform such a power analysis, to extract estimates for the parameter values (e.g., as explained here:^3^).

When recruiting participants, it is important to be transparent about the time investment of ESM research and clearly explain *why* it is helpful to the study to collect data at multiple times during the day rather than just once as is the case in traditional retrospective research. When failing to explain why parents need to fill out the same questions “over and over again,” they may dropout or become frustrated. To increase their compliance, it is also important to accommodate their schedules and talk about how they can fit the ESM into their daily lives. Depending on the study design, it can help to offer multiple starting dates to pick from, suiting their schedule best, instead of one start date for all participants.

### Running an ESM Study

Every researcher wants high-quality data. In ESM research, one can mostly focus on compliance – the percentage of answered questionnaires – as a data quality indicator. Only if compliance is high, one is able to generalize the answers to daily life and data analysis are sufficiently powered. In most studies, participants answer on average 50–95% of all ESM surveys (Wen et al., 2017; van Roekel et al., 2019; Williams et al., 2021). As a rule of thumb, 70–80% compliance indicates good data quality. Answering almost all questionnaires (i.e., compliance close to 100%) can be a sign of reactivity, with participants adapting their daily life to make sure they do not miss a questionnaire. Adapting routines can compromise the ecological validity of an ESM study (Rintala et al., 2019). Nevertheless, whether high compliance compromises an ESM study may depend on the research aim and the length of the study (with longer durations having a higher risk for participants adapting to the questionnaire routine).

To guarantee a sufficient compliance and therefore a good data quality, several steps can be taken. First, by piloting the study, researchers can eradicate technical flaws and adapt the study design to prevent frustration (and potentially resulting in dropout) in their participants (Rintala et al., 2019; Eisele et al., 2020). Second, researchers can explain the importance of filling out most surveys to the participating parents before the study starts. Furthermore, presenting the participants with a manual of the device is associated with higher compliance rates (Morren et al., 2009). In our experience, it is effective when researchers schedule an online or home visit with the participating parents to provide an explanation of the study application or device. Third, participants can be motivated to fill out many surveys with a (financial) reward system that can increase compliance rates (Morren et al., 2009). Note, however, to check within the sample what works as a reward. In our experience, mothers (mostly highly educated) were more motivated to help other parents (by contributing to science) or receive a present for their infant rather than to receive a monetary reward for themselves. In our study, we also added a “fun fact” about babies and/or parenting at the end of each questionnaire, which was indicated as one of the reasons that motivated mothers to continue completion of the daily questionnaires.

Most importantly, during data collection, researchers can ensure a high compliance by monitoring participation. Some software tools offer solutions to keep track of the surveys that are being filled out during the study. Seeing irregularities could be an indication of a technical problem. Therefore, it is advised to include the possibility to contact participants during the study to troubleshoot technical problems or answer questions of participants. Using text-based communications (i.e., purchasing a research-phone) can lower the threshold for participants to contact researchers in case of issues. In their review, Morren et al. (2009) also found that messages from the study researchers were an effective strategy to increase compliance.

### Analyzing Your ESM Dataset

Experience sampling method enables the collection of intensive longitudinal data, with assessments nested within persons (and even within families). When analyzing ESM data, it is important to take into account these nested data structure in order to interpret the results correctly. Additionally, time plays an important role. The data are structured in a long format (vs. the wide data format) due to the multiple time points, with each row representing one time point per participant. Furthermore, for analyses, it is important to consider that assessments on day 1 are likely more strongly related to assessments on day 2 than on day 5 (van Roekel et al., 2019). Multilevel modeling is one analytic strategy that can be used to examine nested data and is often used in ESM studies. While specifying lagged variables is possible in multilevel models to take into account the time-dynamic structure, using software packages designed for examining these lagged dynamic associations is recommended (e.g., Dynamic Structural Equation Modeling (DSEM) in Mplus; (Asparouhov et al., 2018). DSEM is a statistical analysis technique that takes four methods of modeling into consideration that are suitable for ESM-specific data, namely, multilevel modeling, time-dynamic modeling, structural equation modeling, and time-varying effects modeling (Asparouhov et al., 2018). Therefore, DSEM can cater to ESM-specific data by catering to the unique aspects of such a study design and providing a complete picture of the study dynamics (Asparouhov et al., 2018). In addition, ESM studies always have missing data, as 100% compliance is very unlikely (see section compliance). There are several methods to consider missing data in analyses. Therefore, it is important to consider missing data in the analyses of ESM data. For example, for multilevel analyses all available cases can be included, including participants who have not completed every point in time (Bagiella et al., 2000).

In the paper of Keijsers and Van Roekel (2018), suggestions of various methods for analyzing longitudinal data are presented as well as future recommendations, but the field is still under development. Most importantly is that the analyses should align with the research questions and that an adequate plan of analyses is made during the planning phase of the study. A full review of analyses techniques for ESM research is beyond the scope of the current article, and for more information, please see, among others, the studies by Asparouhov et al. (2018), Keijsers and van Roekel (2018), and Vogelsmeier et al. (2021).

## Common Pitfalls and Their Solutions

Like many other types of data collection, collecting data with ESM has its own complications. Because ESM heavily relies on smartphones, many of the pitfalls have to do with or are at least somewhat related to the functioning of these devices or the functioning of the software. Even though the smartphones are quite literally out of our hands, there are still a number of ways in which researchers can avoid some common pitfalls with ESM research, both before and during the study. We discuss these in Box 1 and Box 2, respectively.

BOX 1 | Before the start of your study

✓ Make sure that your study works as you want it to work. It is important to extensively test your study, by a number of people, using smartphones of different brands, makes, and ages
✓ Consider the user experience. What is the length of your questionnaires? How often do you put out questionnaires a day? In short, what is the ESM burden?
  - Carefully consider if ESM burden is also dependent on the answers that your participants give (e.g., if they are alone vs. with others, do they not have to fill out any follow-up questions?). If this is the case, consider whether this is likely to influence the (truthfulness of) responses participants give.
✓ Check that people from your target population understand the questions and that they understand them in the way that you intended them.
✓ Consider your reward scheme; is the reward contingent on filling out a minimum number of questionnaires? Check whether such a scheme can also have negative consequences, for instance by demotivating participants who are not able to meet the minimum due to circumstances.
✓ Consider making a plan of analyses that are most suitable for the data that are going to be collected and to answer the research question appropriately. What are appropriate statistical models for these type of data?

BOX 2 | During the study

✓ Make sure your participants know how to find you when something goes wrong. Consider a low-threshold medium for contact (e.g., SMS, WhatsApp, and call).
✓ Be prepared. Many ESM or daily diary applications will have a forum or help page. Make sure you are well-acquainted with it and already read up on some things that often go wrong.
✓ Keep track of compliance so that you can intervene when you see a participant is becoming less engaged. However, intervene with caution because too much interference can also decrease participantsÃ¢â‚¬â„¢ motivation.

## Conclusion

In sum, ESM is a relatively underused method for investigating infant development, even though it has multiple advantages over other research methods. Besides the possibility it offers to (micro)longitudinally study infant development from a distance, it also enables researchers to collect greatly detailed information about infant development and family dynamics in an ecologically valid manner. Highly dynamic concepts, such as mother-infant interactions, maternal mood, thoughts and behaviors, and infant sleep, cry, and fuss behaviors are particularly suitable for ESM. Nevertheless, transitioning into ESM research can be challenging and requires care, planning, and a commitment to the method – it cannot (and should not) merely be tagged onto existing research (Larson, 2019). With this practical guide, we hope to inform researchers involved in infant development about adding ESM to their research methods and to lower the threshold for incorporating it into their skillset.

## Author Contributions

MH coordinated the writing process, structured the outline, and wrote the introduction and conclusion. AB wrote paragraphs about ESM in general. VH wrote paragraphs about preregistration and participant recruitment and power analyses. LJ wrote paragraphs about wearables. MA wrote paragraphs about advantages and disadvantages. EM wrote paragraphs about common pitfalls and constructed the boxes. MB constructed the reference list. MH and MB monitored writing style, integrated all separate parts, and led the revisions. All authors provided feedback on the first complete draft and reviewed the final version of the manuscript.

## Funding

This project was supported by awards from the Dutch Research Council (NWO), Veni.VI.191G.025 (PI: MH), a Vidi 452-17-011 (PI: Loes Keijsers), a Vici 453-15-006 (PI: Bernet Elzinga), and EM was supported by a grant from the European Research Council (ERC-2017-CoG-773023).

## Conflict of Interest

The authors declare that the research was conducted in the absence of any commercial or financial relationships that could be construed as a potential conflict of interest.

## Publisher’s Note

All claims expressed in this article are solely those of the authors and do not necessarily represent those of their affiliated organizations, or those of the publisher, the editors and the reviewers. Any product that may be evaluated in this article, or claim that may be made by its manufacturer, is not guaranteed or endorsed by the publisher.

## Footnotes

^1^ https://esmitemrepositoryinfo.com/

^2^ https://osf.io/3tznb/

^3^ https://osf.io/2bm6x
